# 

**Published:** 1860-03

**Authors:** 


NORTH AMERICAN

### MEDICO-CHIRURGICAL REVIEW.

Vol. IV.] MARCH, 1860. [No. 2.

### CONTENTS.

### ANALYTICAL AND CRITICAL REVIEWS.

Art. Page

I.—1. American Medical Biography, etc., by James Thacher, M.D.—2. Some Account of the Pennsylvania Hospital.—3. Address to the Medical Graduates of the University of Pennsylvania, by George B. Wood, M.D.—4. General Catalogue of the Medical Graduates of the University of Pennsylvania.—5. Philadelphia and her Medical Schools. —6. An Exposition of the Laws which relate to the Medical Profession in England, by John Davies, M.D. ... 193

II.—A Treatise on Medical Electricity, Theoretical and Practical; its Use in the Treatment of Diseases. By J. Althaus, M.D. ... 242

III.—The Diagnosis, Pathology, and Treatment of the Diseases of the Chest. By W. W. Gerhard, M.D. ... 260

IV.—Gustaf Von Düben's Treatise on Microscopical Diagnosis. By Louis Bauer, M.D. ... 263

V.—An Essay on Hernia. By James Bryan, M.D. ... 265

### ORIGINAL COMMUNICATIONS.

I.—Cases from the Surgical Clinic of the Jefferson Medical College.—Service of Prof. Gross. By Emil Fischer, M.D. ... 266

II.—Cases in Medical and Surgical Practice. By A. Lopez, M.D. ... 285

III.—Proceedings of the Pathological Society of Philadelphia ... 291

### BI-MONTHLY ABSTRACTS.

SURGERY. BY S. W. GROSS, M.D.

Acupressure: A New Method of Arresting Surgical Hemorrhage.—On the Treatment of Strangulated Hernia by the Taxis.—A Case of Simultaneous Lateral Dislocation of both Knees.—Aneurism of the Radial Artery, and of the Crura Artery, cured by Digital Compression.—Review of Cases of Stone in the Bladder. New Methods and Apparatus for Vesico-Vaginal Fistula.—Simultaneous Dislocation of both ends of the Clavicle.—Value of Internal Incision in the Treatment of Obstinate Strictures of the Urethra ... 308

PATHOLOGY AND PRACTICE OF MEDICINE. BY RICHARD J. DUNGLISON, M.D.

Epilepsy, with Ossification of the Falx Cerebri.—Curious Phenomena from the Presence of a Tumor in the Fourth Ventricle of the Brain.—Effect of Cerebellar Hemorrhage on Muscular Motions.—Cerebral Symptoms Independent of Cerebral Disease.—Fatty Degeneration and Acute Softening of the Liver, with Entorrhagia.—Chylous or Milky Urine ... 316

OBSTETRICS, AND DISEASES OF WOMEN AND CHILDREN. BY WM. B. ATKINSON, M.D.

PAGE

Post-Partum Hemorrhage: its Prevention.—Uterine Catheterism with Cat-gut, as a Means of Producing Artificial Premature Labor.—Retroversion of the Uterus in Pregnancy.—Exhaustion after Suckling.—Two Cases of Closure of the Womb Successfully Treated.—On Granulations of the Lining Membrane of the Uterus.—Diagnosis of Ovarian Tumors.—Ovarian Tumor Simulating Pregnancy.—Treatment of the Hysteric Paroxysm by Chloroform.—The Mouth to Mouth *vs.* The Marshall Hall Method in Asphyxia Neonatorum.—Hemorrhage from the Bowel in Children as a Sign of Polypus of the Rectum.—Laryngismus Stridulus .... 324

PHYSIOLOGY. BY S. WEIR MITCHELL, M.D.

Notice of Further Researches on the Coagulation of the Blood.—Experiments as to the Production of Epilepsy in Guinea-pigs.—On the Cause of Death in Animals subjected to High Temperatures.—Presence of Urea in Chyle and Lymph .... 334

MATERIA MEDICA AND THERAPEUTICS; MEDICAL CHEMISTRY; TOXICOLOGY AND LEGAL MEDICINE. BY EMIL FISCHER, M.D.

*Materia Medica and Therapeutics:* On the Dietetic and Medicinal Properties of Erythroxylon Coca.—Injection of Sulphate of Atropine on the Pneumogastric Nerve in Asthma.—A few particulars respecting Woorara.—Curare in Tetanus.—Sambucus Nigri in Dropsy.—Borax in Diphtheritis.—Alum Lozenges in Affections of the Throat.—Phosphorus in Paralysis of the Muscles of the Eyes.—Collyrium in the Ophthalmia of New-born Infants.—Injection of Tincture of Aloes in Gleet.—On the Treatment of Blennorrhagia by Vinum Colchici and Tincture of Opium.—On Chinese Medicines.—Powder for Chronic Coryza.—Garlic and Lemon-juice in Cases of Membranous Angina.—Tannate of Bismuth in Diarrhœa.—Solanaceæ in Tenesmus.—Selinum Palustre in Epilepsy.—Chelidonium Majus in Prurigo.—On the Oil of Aleurites Fribola.—Iodide of Ammonium in the Treatment of Constitutional Syphilis .... 340

*Toxicology and Legal Medicine:* A Case of Emphysema of the Lungs in a Still-born Child.—Fallacy of Sources of Arsenic in Dead Bodies.—Death from Chloroform.—Poisoning by Cyanide of Potassium .... 349

BI-MONTHLY PERISCOPE.

SURGERY.

On the Great Importance of Early Diagnosis and Treatment for Stone in the Bladder.—Antiphlogistic Powers of Morphia, Illustrated by its Use in the Treatment of Acute Inflammations of the Sclerotic and Iris.—On Syphilitic Tumors of the Tongue .... 352

OBSTETRICS.

On Fœtal Auscultation.—A Polypus-shaped Desmoid of nearly Three Pounds Weight extirpated by breaking up .... 364

EDITORS' TABLE.—The Gold-headed Cane. II.—Florence Nightingale.—Private Asylum for the Insane.—Curiosities of French Medical Literature.—Death and Camphene.—Southern Medical Students and Southern Medical Schools.—Inoculability of Diphtheritis.—Hypnotism.—Age of the late Dr. Mütter.—Revision of the United States Pharmacopœia.—The American Medical Association.—Philadelphia Anatomical Rooms.—Jefferson Medical College.—Medical Classes in Canada.—Braithwaite's Retrospect.—New Medical Journals .... 369

NECROLOGICAL NOTICES.—Dr. William F. H. Montgomery.—Dr. Joseph Ayre.—Dr. James Adair Lawrie .... 383

BI-MONTHLY BIBLIOGRAPHICAL RECORD .... 384

